# Severe congenital nephrogenic diabetes insipidus in a compound heterozygote with a new large deletion of the *AQP2* gene. A case report

**DOI:** 10.1002/mgg3.568

**Published:** 2019-02-19

**Authors:** Ramón Peces, Rocío Mena, Carlos Peces, Fernando Santos‐Simarro, Luis Fernández, Sara Afonso, Pablo Lapunzina, Rafael Selgas, Julián Nevado

**Affiliations:** ^1^ Nephrology Department, La Paz University Hospital, IdiPAZ Autonomous University Madrid Spain; ^2^ La Paz University Hospital Medical and Molecular Genetics Institute (INGEMM), IdiPAZ Madrid Spain; ^3^ Basic Research Center in the Rare Diseases Network (CIBERER) Madrid Spain; ^4^ Information Technology Area SESCAM Toledo Spain

**Keywords:** aquaporin 2 gene, compound heterozygous mutation, exonic deletion, nephrogenic diabetes insipidus, p.T125M mutation, polyuria, (SNP) array

## Abstract

**Background:**

Congenital nephrogenic diabetes insipidus (NDI) is a rare condition characterized by severe polyuria, due to the inability of the kidneys to concentrate urine in response to arginine vasopressin (AVP). In the majority of the cases, the disease shows an X‐linked inherited pattern, although an autosomal recessive inheritance was also observed.

**Methods:**

We report a patient with a severe NDI diagnosed during the neonatal period. Because the patient was female without a family history of congenital NDI, her disease was thought to exhibit an autosomal recessive form.

**Results:**

A full mutation analysis of AVP receptor 2 (*AVPR2; *MIM#300538) gene showed no mutations. However, direct Sanger sequencing of the aquaporin 2 (*AQP2*) revealed an apparently homozygous mutation at nucleotide position NM_000486.5:c.374C>T (p.Thr125Met) in exon 2. Further customized multiplex ligation‐dependent probe amplification (MLPA), single‐nucleotide polymorphism (SNP) array analysis, and long‐range polymerase chain reaction (PCR) followed by Sanger sequencing showed a heterozygous exonic deletion comprising exons 2, 3, and partially 4 of *AQP2*.

**Conclusion:**

This is the first case of a compound heterozygote patient with a missense mutation involving NM_000486.5:exon2:c.374C>T (p.Thr125Met) and a gross deletion of at least exons 2, 3, and partially 4 on the *AQP2* to present with a severe NDI phenotype.

## 1 INTRODUCTION

Congenital nephrogenic diabetes insipidus (NDI) is a rare disease that is characterized by the excretion of abnormally large volumes of urine, due to the inability of the kidneys to concentrate urine in response to arginine vasopressin (AVP) (Sasaki, 2004; Wesche, Deen, & Knoers, 2012). Patients with congenital NDI typically present in infancy with severe polyuria, polydipsia, and growth failure. The majority (90%) of inherited cases is caused by mutations in the AVP receptor 2 (*AVPR2; *MIM#300538) gene on chromosome Xq28 (Sasaki, 2004; Wesche et al., 2012). This gene encodes the vasopressin receptor, type 2, also known as the V2 receptor, which belongs to the seven‐transmembrane‐domain G protein‐coupled receptor (GPCR) superfamily, and couples to Gs thus stimulating adenylate cyclase. However, about 10% of congenital NDI might be caused by aquaporin 2 (*AQP2;* MIM#107777) gene mutations, and most of them are autosomal recessive forms of inheritance (Sasaki, 2004; Sasaki, Chiga, Kikuchi, Rai, & Uchida, 2013; Wesche et al., 2012). In fact, the genetic causes of NDI vary among different ethnic groups (Sasaki, 2004; Sasaki et al., 2013; Wesche et al., 2012). The human *AQP2* is located on chromosome 12q13.12 (Sasaki, 2004). This gene has four exons and three introns and encodes a 271‐amino acid protein with six transmembrane domains (Wesche et al., 2012). *AQP2* is expressed predominantly at the apical region of the principal cells of collecting duct and the inner medullary collecting duct cells (Nielsen et al., 1995). In these cells, the vasopressin increases the osmotic water permeability apical membrane by triggering exocytosis of AQP2‐containing vesicles (Nielsen et al., 1995). Here, using several molecular approaches, we report a female proband with NDI to be found a compound heterozygote for a first time reported *AQP2* exonic deletion (comprising at least exons 2, 3, and 4, partially), and also carrying a missense mutation, previously described in the undeleted allele.

## 2 METHODS

### 2.1 Case presentation

The patient, currently 32‐year‐old, was the second child of a healthy nonconsanguineous couple. Family history was negative for NDI disease. The pregnancy was uneventful, and the child was delivered to term by vaginal via. Birth weight was 3,300 g. Her elder brother was healthy. She was admitted to the hospital at the age of 4 weeks presenting symptoms of dehydration, polyuria, polydipsia, vomiting, fever of unknown origin, and weight loss. Laboratory data showed hypernatremia, high serum osmolality, and low urine osmolality. Thus, the diagnosis of NDI was suspected. Treatment with adequate hydration and hydrochlorothiazide initiated 2 days later leads to ion levels normalized, as well as blood urea and serum creatinine levels. Clinical diagnosis was confirmed by dDAVP infusion test without elevation of urine osmolality (before 58 mOsmol/kg H_2_O and after 69 mOsmol/kg H_2_O). Thereafter, she developed similar episodes of vomiting and febrile illness requiring hospitalization five more times up to the last episode, produced in 1998. At the age of 22 years was remitted to our out‐patient clinic for a complete clinical evaluation. Brain magnetic resonance imaging and ultrasonographic examination of both kidneys revealed no abnormality at all. Currently (with 32 years old), her height is 158 cm and her weight 48 kg. She remains with stable polyuria of about 6–6.5 L/day and maintains normal serum levels of sodium, potassium, calcium, bicarbonate, and phosphate, with hypocalciuria (urine calcium/creatinine, 0.1 mg/mg), a serum level of magnesium of 1.6 mg/dl with a fractional urinary excretion of magnesium of 13%, a blood urea of 23 mg/dl, a serum creatinine of 0.75 mg/dl, and MDRD of 109 ml/min/1.73 m^2^. Urine osmolality and plasma osmolality are 138 and 285 mOsm/Kg H_2_O, respectively. The treatment currently is with hydrochlorothiazide (1.5 mg/kg per day), amiloride (0.15 mg/kg per day), indomethacin (100 mg per day), potassium chloride (40 mmol per day), magnesium hydroxide (308 mg per day), and ranitidine (300 mg per day). Recently, she is also under treatment with fluvastatin (40 mg daily).

### 2.2 Molecular analysis

#### 2.2.1 Sanger direct sequencing for AVPR2 and AQP2

Genomic DNA was extracted from peripheral blood lymphocytes with Gentra Puregene Blood Kit (QIAGEN GmbH, Hilden, Germany). Genomic DNA mutation screening of the *AVPR2 *and *AQP2 *genes was performed for our proband and her parents. New primers were designed to amplify all exons and flanking intronic regions of both genes, so that all fragments could be amplified by polymerase chain reaction (PCR) simultaneously. After amplification, the PCR products were purified using a PCR purification kit (GE Healthcare) and then sequenced with BigDye 3.1 (Applied Biosystems; life technologies). The sequences were analyzed with an ABI PRISM 37130xl DNA Analyzer (Applied Biosystems Life Technologies, USA).

#### 2.2.2 Short tandem repeats (STR) microsatellites analyses

A panel of eight microsatellites corresponding to chromosome 12 was evaluated in the propositus and her parents (the complete list of STRs under request). Sequences of the primers were obtained from the NCBI public database (http://www.ncbi.nlm.nih.gov/sites/entrez). PCR and capillary electrophoresis conditions were adapted from universal laboratory protocols.

#### 2.2.3 Multiplex ligation‐dependent probe amplification (MLPA) analysis

A customized MLPA was designed by us. Briefly, four synthetic DNA probes within *AQP2* plus four reference probes were designed, complementary to the regions we wanted to test. Targets were located in the 5′ upstream region and intronic regions 1, 2, and 3 of the gene, whereas reference probes hybridized to other chromosomal regions outside of 12q13.12, expected to be normal. Following amplification and fragment separation in an automated DNA sequence analyzer (ABI3130XL; Life Tech. Grand Island, NY, USA). Data analysis was made against up to 5 control samples using than in‐house Excel spreadsheet. Target peak heights were normalized against the sum of all control peak heights, using this value to express the *AQP2* dosage as a patient sample/control sample ratio (1 = normal, 0.5 = deletion, 1.5 = duplication).

#### 2.2.4 High‐density (SNP) arrays

Extracted DNAs were quantified using PicoGreen (Invitrogen Corporation, Carlsbad, CA). A genome‐wide scan of 850,00 tag SNPs was conducted on the proband, using the Illumina CytoSNP‐850k BeadChip according to the manufacturer's specifications (Illumina, San Diego, CA). GenCall scores < 0.15 at any locus were considered "no calls." Image data were analyzed using the Chromosome Viewer tool contained in Genome Studio (Illumina, San Diego, CA). The metric used was the log R ratio which is the log (base 2) ratio of the observed normalized *R* value for a SNP divided by the expected normalized *R* value (under manufacturer`s specifications). In addition, an allele frequency analysis was applied for all SNPs. All genomic positions were based upon NCBI Build 37 (dbSNP version 130).

#### 2.2.5 Detection of breakpoints and junction fragment analysis with long‐range PCR and sanger sequencing

To precisely determine the sequence at the breakpoints of the exonic *AQP2* deletion, we designed a different set of primers (available upon request) located immediately outside the deleted region, according to our (SNP) array results. The PCRs were performed according to previous described methods. Sequence chromatograms were aligned and analyzed by Sequencher version‐4.8 (Gene Code, Applied Biosciences; http//www.genecodes.com). Whole junction fragments were analyzed with BLASTN application (BLASTN application; http//www.ncbi.nlm.nih.gob/BLAST), University of California Santa Cruz Genome Browser (https://genome.ucsc.edu/), and RepeatMasker application; http://www.repeatmasker.org/).

## 3 RESULTS

**Figure 1:**
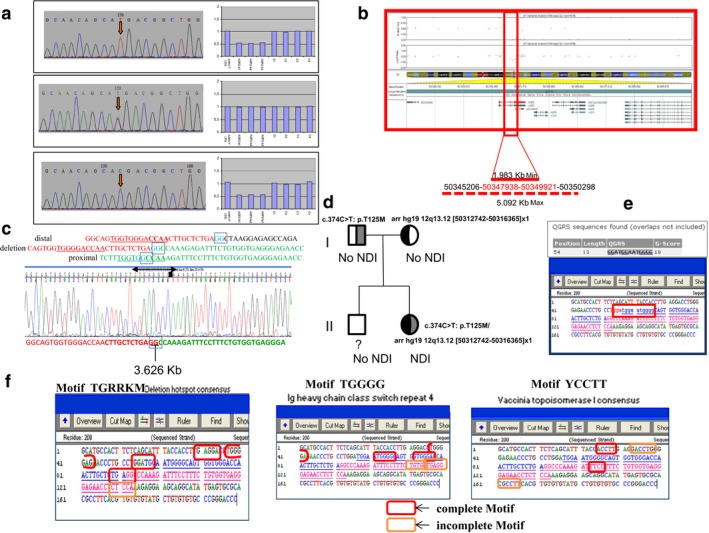
(a) Left panel: sequence electropherograms of *AQP2* exon 2, showing the nucleotide change NM_000486.5:exon2:c.374C>T (p.Thr125Met) (red arrow) in the proband (upper panel), her father (middle panel), and the wild‐type sequence in her mother (bottom panel). Right panel: MLPA analysis showing *AQP2* dosage of the proband (up), her father (middle), and her mother (down). C: control sample; In: intron; UTR: untranslated region. (b) (SNP) array analysis in the proband using CytoSNP 850K (Illumina, USA). Allele frequency and log R ratio of the SNPs within *AQP2* in chromosome 12 have shown. (c) Delimitation of breakpoints for deletion at *AQP2* region using specific primers (available upon request), long‐range PCR, and Sanger sequencing in an ABI 3070 XL. (d) Mutation pedigree in the family. (e) Predicted G‐quadruplex sequences in the patient included herein using QGRS software. Analysis was performed within 100 bp at the breakpoint site. (f) Overview of sequence motifs at the delineated breakpoints. Whole junction fragment analysis was performed with BLASTN application, University of California Santa Cruz Genome Browser, Sequencher DNA Sequence Analysis Software, and RepeatMasker application

After obtaining written informed consent, punctual mutation analysis of the patient and her parents was initially performed by PCR amplification and direct DNA sequencing of the *AVPR2 *using a set of newly designed primers (available upon request). Full mutational analysis of the *AVPR2 *demonstrated no mutations in the proband neither her parents. However, analysis of the *AQP2 *(primers were available upon request) revealed an, apparently, homozygous mutation NM_000486.5:exon2:c.374C>T (p.Thr125Met). Parental analysis revealed that her father was heterozygous for the same missense mutation (Figure 1a, middle left panel), whereas no punctual mutation in *AQP2* was found in her mother (Figure 1a, bottom left panel). This discordant allelic segregation in our proband could be explained by either an *AQP2* deletion in the other allele (inherited from her mother), causing a loss of heterozygosity and an apparently homozygous mutation in exon 2, or consequence of an uniparental disomy (UPD) of chromosome 12. A segregation study of eight microsatellite markers flanking *AQP2* ruled out UPD at this chromosomal region (data not shown), as well as the (SNP) array in the proband after all (Figure 1b).

A customized MLPA panel was designed by us to test a putative *AQP2* deletion in the other allele (see Methods). Results confirmed a chromosomal deletion in the patient and her mother, spanning minimally from intron 1 to intron 3 and thus affecting at least, exons 2 and 3 of *AQP2 *(Figure 1a, top and bottom right panels). The expected size for the deletion was estimated of at least 1.27 kb (genomic coordinates; [hg19]: 50347770_50349044), and no estimated distal breakpoint can be done using MLPA. Additional (SNP) array analysis (cytoSNP850K, Illumina) in the proband established the size of deletion between 1,983 and 5,092 kb (see Figure 1b; genomic coordinates; [hg19]: 50345206_50350298 and [hg19]: 50347938_50349021, respectively) within exon 2 to exon 4, partially. In addition, based on (SNP) array genomic coordinates, we designed primers to determine deletion breakpoints by a long‐range PCR, which is showed in Figure 1c, establishing a deletion of 3.6 kb long. The brother of the patient (who has no renal affectation) did not undergo any molecular genetic analysis yet (Figure 1d).

### 3.1 Use of chemical chaperones

Finally, during the last 12 months, she was under treatment with fluvastatin (40 mg/daily), which was not accompanied by a reduction of diuresis or an increase of urine osmolality.

## 4 DISCUSSION

**Figure 2:**
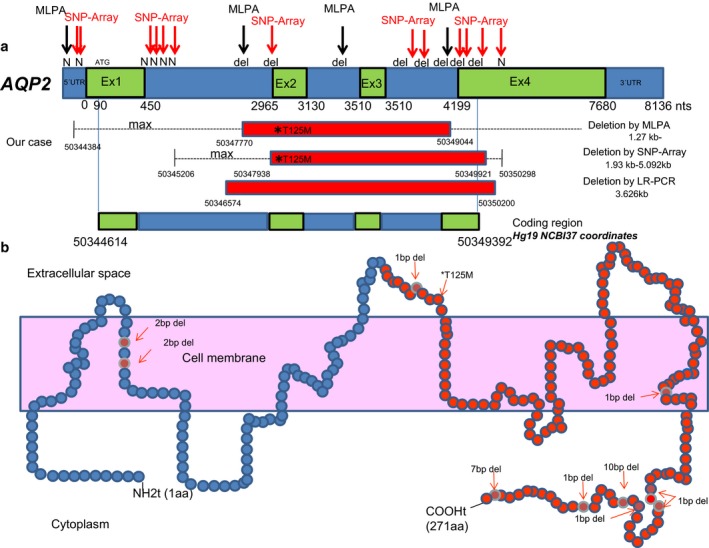
Schematic representation of mutations found in the proband within *AQP2 *using different technologies. (a) Structure of aquaporin 2 embedded in the cell membrane reporting the protein mutations by deletion that cause nephrogenic diabetes insipidus (in blue). (b) The location of the identified deletion of at least exons 2, 3, and 4 is marked in red in the AQP2 protein

AQP2, located at the luminal side of the collecting duct principal cells, is a water channel responsible for the final concentration of urine in response to AVP. Lack of function, often occurring through mistargeting of mutated proteins, induces NDI, a condition characterized by large urinary volumes. Autosomal recessive NDI is a rare disease usually seen in patient with consanguineous parents (Bichet et al., 2012; Sasaki, 2004; Wesche et al., 2012). Since most AQP2‐related NDI are found to be recessive hereditary traits (Robben, Knoers, & Deen, 2006), heterozygotes bearing one wild‐type form of the protein are usually nonsymptomatic as two defective alleles are required to induce the disease. We report on a case of severe autosomal recessive NDI in a female patient with nonconsanguineous parents. Severe polyuria and polydipsia began soon after birth, and these findings in association with the patient's sex and the failure of dDAVP to relieve symptoms suggested that NDI could be caused by mutation(s) in the *AQP2*. In fact, we found a compound heterozygous mutation, NM_000486.5:c.374C>T (p.Thr125Met) of exon 2, which have been previously described (Goji et al., 1998; Kuwahara, 1998; Kuwahara et al., 2001; Marr, Bichet, Hoefs et al., 2002; Shinbo et al., 1999; Tsutsumi et al., 2009) and a deletion of at least exons 2, 3, and partially 4, which has not been described previously, in the *AQP2* of our proband (3.6 kb long; Figures 1c and 2a). Haploinsufficiency score for *AQP2* was <50% (43.8%, which means medium to high score of haploinsufficiency) (Huang, Lee, Marcotte, & Hurles, 2010). The *AQP2* point mutation NM_000486.5:exon2:c.374C>T (p.Thr125Met) was inherited from the asymptomatic father, while her mother carried the exonic deletion, and neither showed any manifestations of NDI disease. In fact, clinical ion level analysis either in blood or in urine, as well as the osmolality monitoring in both parents revealed normal values without suspicious NDI disease. In addition, her elder brother had no NDI either. Thus, in this family the two combined mutations (Figure 1d) seem to be disease causative for a severe clinical form of NDI.

So far, various mutations in the *AQP2 *have been demonstrated in both the autosomal recessive and dominant forms of NDI, in which mutations are distributed throughout the gene without any apparently mutation hot spot (Moon et al., 2009; Sasaki, 2004; Sasaki et al., 2013; Wesche et al., 2012). Currently, 54 mutations known to give rise to autosomal recessive NDI (Milano, Carmosino, Gerbino, Svelto, & Procino, 2017; Moeller, Rittig, & Fenton, 2013; Wesche et al., 2012). Thus, 46 missense/nonsense, four splicing mutations, and one small insertion were reported. In addition, a minority of cases (up to seven), NDI results from small genomic deletions in the *AQP2 *(Bichet et al., 2012; García Castaño et al., 2015; Lieburg et al., 1994; Marr, Bichet, Lonergan et al., 2002; Moeller et al., 2013; Ohzeki, Igarashi, & Okamoto, 1984; Tajima, Okuhara, Satoh, Nakae, & Fujieda, 2003). However, no gross deletion was found at this time so ever: a deletion of G at nucleotide 721 (721delG), a deletion of 10 nucleotides starting at nucleotide 763 (763_772del), and a deletion of 7 nucleotides starting at nucleotide 812 (812_818del) (Bichet et al., 2012; Kuwahara et al., 2001; Sohara et al., 2006). In addition, a study by Park, Baik, Cheong, and Kang, (2014) described a young Korean male with a compound heterozygous mutations located in exon 1. One of the mutations was a frameshift mutation resulting from a deletion NM_000486.5:c.127_128delCA (p.Gln43Aspfs*63). Cen, Nie, Duan, and Gu, (2015) reported two Chinese male individuals with inherited NDI. Proband 1 presented the previously reported heterozygous frameshift mutation c.127_128delCA (p.Gln43Aspfs*63) inherited from the mother and a novel frameshift mutation c.501_502insC (p.Val168Argfs*30), inherited from the father, suggesting that the patient with truncated AQP2 protein presented with much more severe NDI manifestations.

The relationships between *AQP2* mutations and its function have not been completely elucidated. However, misfolding of the protein and retention in the endoplasmic reticulum have been reported in most *AQP2* mutations (Cheong et al., 2005), except for those of NM_000486.5:exon2:c.374C>T (p.Thr125Met), in which a disrupted water channel function is indicated (Goji et al., 1998; Marr, Bichet, Hoefs et al., 2002). To date, this missense mutation in the *AQP2* has been described in several cases, the majority in Japanese families (Goji et al., 1998; Kuwahara, 1998; Kuwahara et al., 2001; Marr, Bichet, Hoefs et al., 2002; Tsutsumi et al., 2009), yielding a protein to be partially functional water channel (retained 25% of the single‐channel permeability) (Marr, Bichet, Hoefs et al., 2002). Moreover, NM_000486.5:exon2:c.374C>T (p.Thr125Met) mutation in the *AQP2* has been also reported in several cases of NDI that were compound heterozygotes and were severely affected (Boussemart, Nsota, Martin‐Coignard, & Champion, 2009; Goji et al., 1998; Iolascon et al., 2007; Kuwahara, 1998; Leduc‐Nadeau et al., 2010; Lieburg et al., 1994; Tsutsumi et al., 2009), as the case described herein. These findings suggest that in these cases the resulted truncated protein cannot exert its proper water channel function. As far as we know, a compound heterozygous for a punctual mutation and a gross deletion has not been reported to date. It is noteworthy the two mutations may produce very different effects on AQP2 protein, a loss of channel function in the case of this missense mutation, and a severely altered AQP2 structure in the case of deletion. By analogy with other autosomal recessive disorders, one can predict that the combination of mutations presented in each allele may also play a role in the phenotype variability and severity of NDI. On the other hand, 7 small deletions have been previously found in affected with severe features of NDI disease (Bichet et al., 2012; Sasaki et al., 2013). We described herein a novel exonic deletion (including exons 2, 3, and partially 4) as a recessive trait in a patient showing a severe phenotype of NDI. This new deletion is within a preserved region of *AQP2 *that comprises several transmembrane domains of the protein, which are highly conserved among species (Sasaki et al., 2013). Deletions affecting exon 1 and other small deletions placed in the C‐terminus of *AQP2 *(all of this with a dominant inheritance) were also associated with a more severe form of the disease than mutations in other parts of the gene (Kuwahara et al., 2001; Sasaki et al., 2013; Sohara et al., 2006). Interestingly, the gross deletion described herein (affecting more than half of the protein, including C‐terminus, with an intact exon1) may also have a severe clinical phenotype and supports a critical role of the C‐terminus of the AQP2 in the functional trafficking of this protein. However, it did not show a dominant inheritance, because of the strict monitoring of ion levels in the carrier asymptomatic mother.

We characterized the breakpoints in the deletion by using the PCR mapping and junction fragment sequencing, followed by Bio‐informatic analysis in both breakpoints through several web tools, showing that those lay next to highly homologous repetitive sequences of two SINE elements (MIR type), but they did not mediate the rearrangements directly. According to the UCSC Genome Browser, the region deleted was not flanked by segmental duplications, although repeat element analysis demonstrated that deleted region was flanked by 2 bp (GG) stretch of identical sequence (Figure 1c) that may point out to a nonreplicative mechanism of formation, such as is nonhomologous end joining (NHEJ) (Lieber, 2008). In addition, it also revealed several repeat and genomic architectural elements, such as palindromic DNA or stem‐loop structures within the deleted breakpoints that may modulate this nonrecurrent genomic rearrangement (Figure 1e–f). Searching for more than 20 specific sequence motifs (involved in DNA rearrangements elsewhere) in the 50 bp centered on the breakpoints led to the identification of a total of three consensus patterns (Figure 1f); among them, an immunoglobulin class switch repeat is found. The presence of this switch region motif, such as in several large *AVPR2* deletions (Vargas‐Poussou et al., 1997), is therefore consistent with an involvement of these sequences in promoting these rearrangements. Finally, the deletion may also create a possible deletion hot spot at the breakpoints (Figure 1f), and moreover, a predicted G‐quadruplex sequence “GAATGGAATGGGG” was also found at 5′end of the deletion (Figure 1e).

Our patient was diagnosed with NDI on the basis of typical neonatal manifestations. Therapeutically, she received treatment with hydrochlorothiazide, amiloride, and indomethacin (Anesi, Gemmis, Galla, & Hladnik, 2012; Bichet et al., 2012; Olesen, Rutzler, Moeller, Praetorius, & Fenton, 2011; Wesche et al., 2012). Moreover, she received potassium, magnesium, and ranitidine. Despite which, she maintained polyuria of about 6 L/day. Indeed, this patient had a severe form of NDI. Recently, chemical chaperones have been tested to correct aberrant folding of AQP2 in autosomal recessive NDI (Moeller et al., 2013). Based on this, we analyzed the effect of treatment with fluvastatin during 12 months without significant changes in urine excretion of water, sodium, potassium, and chloride. Therefore, although a possible therapeutic approach to rescue the plasma membrane expression of functional misfolded mutant proteins is the use of chemical chaperones to promote escape from the endoplasmic reticulum, for *AQP2* mutations caused by deletions, insertions, splicing, or other complex rearrangements, it is impossible to consider this therapeutic option (Moeller et al., 2013).

In summary, this is the first case of NDI due to a compound heterozygous mutation; a novel exonic deletion within at least exons 2, 3, and 4 (for a segment of 3.62 kb) of *AQP2*, and a previously reported point mutation NM_000486.5:exon2:c.374C>T (p.Thr125Met). This study provides significant evidence that the interaction of both mutations is likely to be responsible for the severity of NDI symptoms in this female patient. It also emphasizes that the restriction of the identification of a single genetic defect can be misleading. The presence of additional gene mutations should be considered, especially if there is familial discordant allelic segregation. In addition, this novel exonic deletion as a recessive trait increases the spectrum type of the mutations affecting *AQP2*.

## ETHICS APPROVAL AND CONSENT TO PARTICIPATE

This article was conducted in accordance with the World Medical Association Declaration of Helsinki, all its amendments and national regulations.

## CONSENT FOR PUBLICATION

Written informed consents were obtained from the patients for publication of this article.

## AVAILABILITY OF DATA AND MATERIAL

The dataset supporting the conclusions of this article is included within the article.

## CONFLICTS OF INTEREST

The authors declare that there is no conflict of interest regarding the publication of this paper.

## AUTHORS’ CONTRIBUTIONS

All authors were involved in drafting the manuscript, gave final approval for the version to be published, and agreed to be accountable for all aspects of the work in ensuring that questions related to the accuracy or integrity of any part of the work are appropriately investigated and resolved. RP made substantial contributions to the concept/design and acquisition, analysis, and interpretation of data. RM, CP, SA, PL, LF, FSS, RS, and JN made substantial contributions to the acquisition, analysis, and interpretation of data.
